# Histomorphology, ultrastructure and fatty acid composition of the adipose tissue in pansteatitis, the potentials in understanding the underlying mechanism and diagnosis of pansteatitis in the Nile crocodile

**DOI:** 10.1186/s12944-016-0405-2

**Published:** 2017-02-23

**Authors:** O. I. Azeez, J. G. Myburgh, R. A. Meintjes, M. C. Oosthuizen, J. P. Chamunorwa

**Affiliations:** 1 Anatomy and Physiology Department, Faculty of Veterinary Science, University of Pretoria, Onderstepoort 0110, Pretoria, South Africa; 2 Veterinary Tropical Diseases Department, Faculty of Veterinary Science, University of Pretoria, Onderstepoort 0110, Pretoria, South Africa; 3 Paraclinical Science Department, Faculty of Veterinary Science, University of Pretoria, Onderstepoort 0110, Pretoria, South Africa; 4 Department of Veterinary Physiology, Biochemistry and Pharmacology, University of Ibadan, Ibadan, Nigeria

**Keywords:** Adipose tissue, Histomorphology, Long chain fatty acid, Pansteatitis, Nile crocodile

## Abstract

**Background:**

In an effort to characterize the fat body and other adipose tissue in the Nile crocodile and the effects of pansteatitis on the structure and composition of the adipose tissue, we evaluated the regional variation in structure and fatty acid composition of healthy farmed crocodiles and those affected by pansteatitis.

**Methods:**

Adipose tissue samples were collected from the subcutaneous, visceral and intramuscular fat and the abdominal fat body of ten 4-year old juvenile crocodiles from Izinthaba Crocodile Farm, Pretoria, South Africa while pansteatitis samples were collected from visceral and intramuscular fat of crocodiles that had died of pansteatitis at the Olifant River, Mpumalanga, also in South Africa. Histomorphology, ultrastrustucture and fatty acid composition by fatty acid methyl ester (FAME) analysis were conducted.

**Results:**

Histological examination showed regional variations in the adipose tissue especially in the collagen content of the ECM, tissue perfusion and division into lobes and lobules by fibrous capsule. Considerable fibrosis, mononuclear cell infiltration especially by macrophages and lymphocytes and toxic changes in the nucleus were observed in the pansteatitis samples.

Regional variation in lipid composition especially in Myristoleic (C14:1), Erucic acid (C22:1n9), and Docosadienoic acid (C22:2n6) was observed. Most of the saturated and trans fatty acids were found in significant quantities in the pansteatitis samples, but had very low levels of the cis fatty acid and the essential fatty acids with C18 backbone.

**Conclusion:**

This study demonstrates that there exists some regional variation in histomorphology and fatty acid composition in the healthy adipose tissue of the Nile crocodile. It also showed that pansteatitis in the Nile crocodile might have been triggered by sudden change in energy balance from consumption of dead fish; and probable exposure to toxic environmental conditions with the evidence of up scaled monounsaturated long chain fatty acids composition and toxic changes in the leucocytes observed in pansteatitis in the present study.

## Background

The quest to unravel the mechanisms behind pansteatitis (inflammation of adipose tissue) and crocodile die offs in the Olifants River and Loskop Dam in Mpumalanga province of South Africa is still ongoing. Several suggestions have been made as predisposing factors and probable aetiology, including consumption of fish that had previously died as a result of environmental pollution of the Olifants River and its tributaries [24, 27]. The river drains some of the industrial effluents, agricultural runoff water and human sewage as well as acid mine drainage (AMD) water from abandoned coal mines around Middleburg Colliery, Witbank [3]. Others have also suggested that there is an association between vitamin E deficiency and pansteatitis following excessive consumption of unsaturated fatty acid or oxidized fat that could deplete vitamin E [12]. It is believed that lack of vitamin E or other antioxidants may predispose the animals to accumulated reactive oxygen radicals and lipid peroxidation [28].

In a further study on the probable direct impact of environmental pollution and heavy metals from AMD waters which seeps into Olifants River from Blesboak stream at a pH of 2.1 on the pathogenesis of pansteatitis, Oberholster et al. [27] reported an association between accumulation of heavy metals especially aluminium and iron and development of yellow fats in Oreochromis mossambicus (Tilapia fish) and bioaccumulation of Al and Fe in filamentous algae, Spirogyra fluviatilis and S. adanata that are often consumed by the fish. They suggested that the yellowness of the fat in O. mossambicus might be as a result of membrane lipid peroxidation by the pro-oxidant action of aluminium as previously suggested by Yoshino et al. [36].

Adipose tissues in vertebrates generally are storage sites for lipids for release of energy via lipolysis to acyl-CoA, β-oxidation to acetyl-CoA for energy production during fasting, starvation or hibernation and estivation in some animals. It has also been noted to be an endocrine organ producing leptin and adiponectin; several inflammatory cytokines and renin-angiotensin system [19]; nutrient and energy sensing and mediator of inflammation and immune cells signalling [17, 29]. The structure and composition is also variable as it undergoes constant remodelling, adapting the cell size and numbers to nutrient availability and hormonal influences as has been studied in humans [33]. Despite the role of adipose tissue in inflammation, generalized inflammation involving the adipose tissues is not a common occurrence and quite difficult to reproduce. It has also been reported that regional variation in structure of white adipose tissue in human is also responsible for their activities and inflammatory cytokines. For example, visceral adipose tissue from the mesenteric, omental and retroperitoneal fat produce more pro-inflammatory cytokines while the subcutaneous fats produces more leptin and adiponectin [33]. Even subcutaneous fats show some degree of regional variation in structure, composition and functions [30].

This therefore calls for another outlook on the structure and composition of the adipose tissues in the Nile crocodile in healthy tissues and during pansteatitis for better understanding of the factors that might have predisposed the tissue to pansteatitis, and determine which of the fatty acid component might be affected in the in vivo esterification/peroxidation that might be responsible for the hardening of all adipose tissue in the affected crocodiles. Previous study on the lipid properties of crocodiles affected by pansteatitis [28] did not take cognizance of possible regional variation in the structure, lipid composition as it affects the release of mediators of inflammation by adipose tissue.

This part of the study is therefore aimed at evaluating the histology, ultrastructure and lipid composition of the adipose tissue of the Nile crocodile from various regions of the body in healthy farm bred crocodiles, compared with pansteatitis samples from crocodiles that had died of pansteatitis.

## Methods

### Sample collection

Fourty adipose tissue samples were collected from the subcutaneous, viscera (omental fat around the stomach and the liver), abdominal fat body, and intramuscular fat from between the muscles in the tail, and ten liver samples, from ten randomly selected, 4 years old juvenile Nile crocodiles. The samples were collected from Izinthaba Crocodile Farm (Pty) Ltd, Farm 59 (435JQ) Vissershoek, De Wildt area, Pretoria, South Africa. Ten pansteatitis samples from the visceral and intramuscular adipose tissues were collected from frozen adipose tissue and body parts of Nile crocodiles that had died of pansteatitis.

Samples for histology and ultrastructural studies were collected into 10% neutral buffered formalin (10% formalin in 0.08 M Sodium phosphate at pH 7.4) while samples for lipid profile analysis were collected into homogenizing buffer consisting of 50 mM Tris–HCl, 1.15% KCl at pH 7.4 in distilled water and stored frozen until the analysis time.

### Histomorphometry

Samples were prepared for histology using haematoxylin and eosin (H and E) stain by fixation in 10% buffered formalin, dehydration in graded alcohol and embedded in paraffin at 60 °C inside labelled embedding mould or cassettes. The embedded tissues were then sectioned using a microtome at 5 μm thickness and each section floated in 45 °C water bath to allow crinkled part to spread before being floated on the glass microscope slide for proper adherence unto the glass slide. The slides were stained with haematoxylin and counterstained with eosin using standard protocol. Slides were examined using Olympus BX 63 light microscope (Olympus Corporation, Tokyo, Japan) at × 10, × 40 and × 100 (in oil immersion) magnifications.

### Transmission electron microscopy

Paraffin embedded tissue blocks previously used for histology were used for the transmission electron microscope study. Samples were cleared with propylene oxide for 20 minutes and embedded in epoxy resin (TAAB 812 resin; TAAB Laboratories, Berkshire, England). Thin sections were cut with Reichert-Jung Ultracut (C. Reichert AG, Vienna, Austria) ultra-microtome using a diamond knife, and stained with lead citrate and uranyl acetate as previously described [10]. TEM viewing was done on Philips CM10 transmission electron microscope at 80 kV (Philips Electron Optical Division, Eindhoven, Netherlands).

### Long chain fatty acid analysis

Fatty acids are obtained by a saponification procedure [6] with modifications [7] while methyl esters are prepared by the addition of 14% Boron triflouride-methanol (BF3/CH3OH).Fatty acids were identified using a Shimadzu Tracera GC 2010 with Restek 2560 (fused silica) column. Column temperature was 140–240 °C, while injector temperature was at 250 °C and BID (barrier discharge ionization detector) at 280 °C. The carrier gas employed by the instrument was Helium, at linear velocity flow rate of 25 cm/sec. Fatty acids were identified by comparison with the retention times of fatty Acid methyl ester peaks (Supelco 37 Fame mix standard, Sigma Aldrich, USA). Calibration and quantification of results was done using LabSolutions Software, results taken as quantified by the software in mg/dL and percentage of the total fatty acid composition.

### Statistical analysis

Values are presented as means ± sem. Mean values of the fatty acid composition were compared by One-way ANOVA, with Tukey’s post-test for multiple comparisons between groups, using GraphPad Prism statistical software, version 5.01 for Windows, GraphPad Software, San Diego California USA (www.graphpad.com). A probability value of 0.05 and below was taken to be significant.

## Results

### Histology

Samples of adipose tissue that were taken from different regions were evaluated histologically using H and E. The samples included the abdominal fat body visceral, intramuscular and subcutaneous fats.

### Visceral adipose tissue

**Fig. 1:**
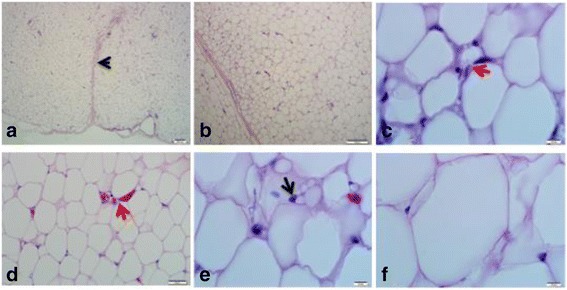
Histology (H and E) of the visceral adipose tissue of the Nile crocodile (*Crocodylus niloticus*). Note the fibrous connective tissue capsule in a (black arrow head), systematic increase in size of adipocytes from the capsule (**b**), ECM (red arrow) with collagen, inflammatory cells and blood capillaries (**c** and **d**), immature adipocytes with dispersed chromatin and prominent nucleolus (black arrow in **c**) and a mature adipocyte with lipid vacuole and displaced nucleus (**f**). Bars show magnifications at scales **a**, 200 μm, **b**, 100 μm, **c** – **f**, 10 μm

The adipose from this region including the omental and mesenteric fat is bounded by a thick fibrous connective tissue capsule that is made up of collagen and fibroblasts, which tends to extend deep into the parenchyma to divide the tissue into lobes (Fig. 1a). Major blood vessels are seen in pairs with an artery in close proximity to its draining vein, close to the capsule. The adipocytes appear hexagonal in shape with relatively uniform sizes of which appear to increase gradually and systematically as the cells get farther away from the capsule and the major blood vessels (Fig. 1b). The cells are filled with a single vacuole of lipids that takes about 98% of the cell volume. The nucleus is displaced to the periphery, flattened and elongated with thick dense chromatin as the lipid vacuole pushes it to the periphery. The extracellular matrix appears filled with thick dense collagen materials scattered blood capillaries, immature adipocytes, fibroblasts and some inflammatory cells especially small lymphocytes and macrophages (Fig. 1c and d). The ECM also gives the tissue some sense of looseness because of its wideness.

### Subcutaneous adipose tissue

**Fig. 2:**
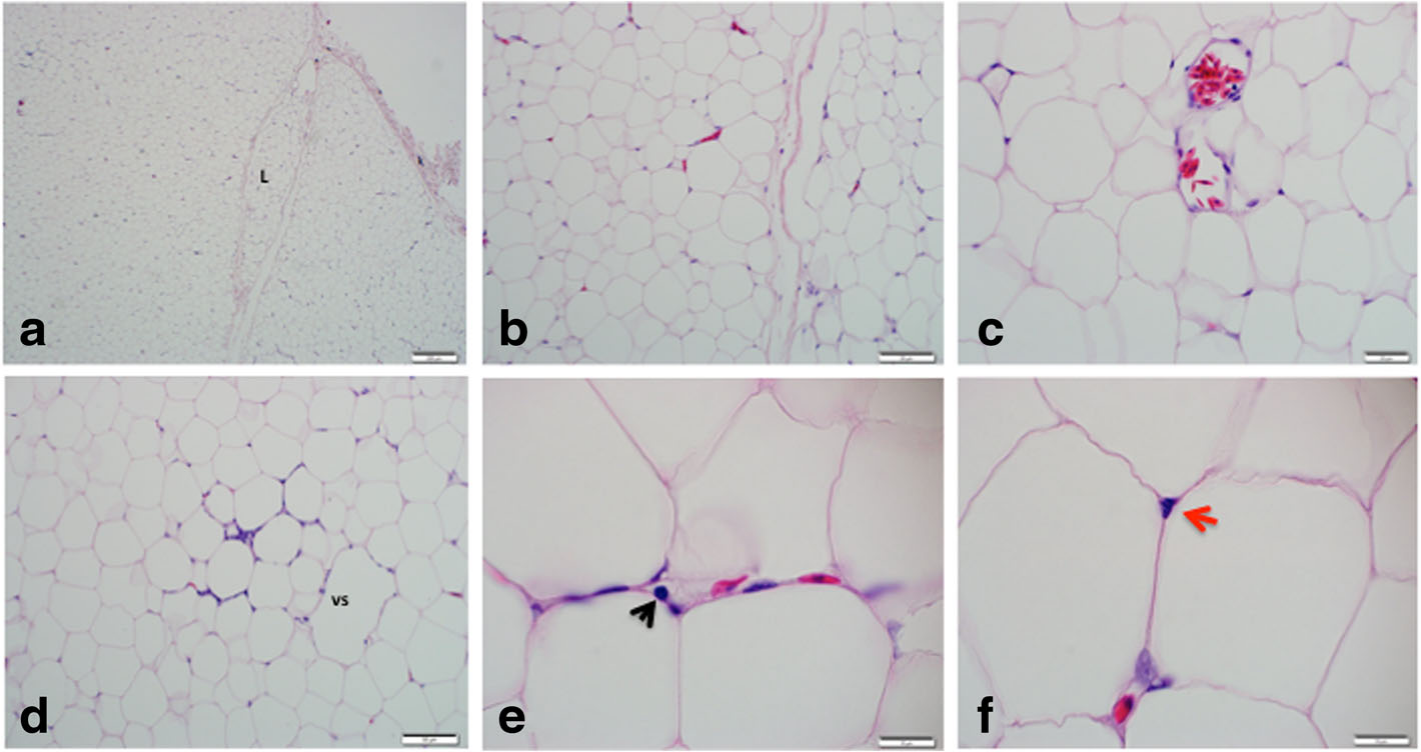
Histology (Hand E) of the subcutaneous adipose tissue of the Nile crocodile (*Niloticus crocodylus*) showing small lobe, L bounded by fibrous connective tissue **a**, round to hexagonal shaped adipocytes with variable sizes **b**, blood vessels and venous sinusoid (vs) in **c** and **d**, inflammatory cell (black arrow) and pyknotic nucleus (red arrow). Bars show the magnifications at scales **a**, 200 μm, **b**, 50 μm, **c**, 20 μm, **d**, 50 μm, **e** and **f**, 10 μm

The subcutaneous adipose tissue is also bound by collagenous fibrous connective tissue capsule that often divide the tissue into small lobes (Fig.2a) unlike the capsule in the visceral adipose tissue that only extends into the parenchyma. The adipose tissue consists of more arteries and veins located deep within the parenchyma and some venous sinusoids (Fig.2d). This is also different from those in the visceral adipose tissue where most arteries are located in the periphery, close to the capsule. The adipocytes are round to hexagonal in shape but with less uniformity in size, and they are more tightly packed with less ECM spaces. Collagen fibres and fibroblasts also appear less in the subcutaneous adipose tissue than in the visceral fat. Also observed are lipid filled adipocytes with pyknotic nuclei, showing shrunken nuclei with dark highly condensed nuclear chromatin (Fig. 2f).

### Abdominal fat body

**Fig. 3:**
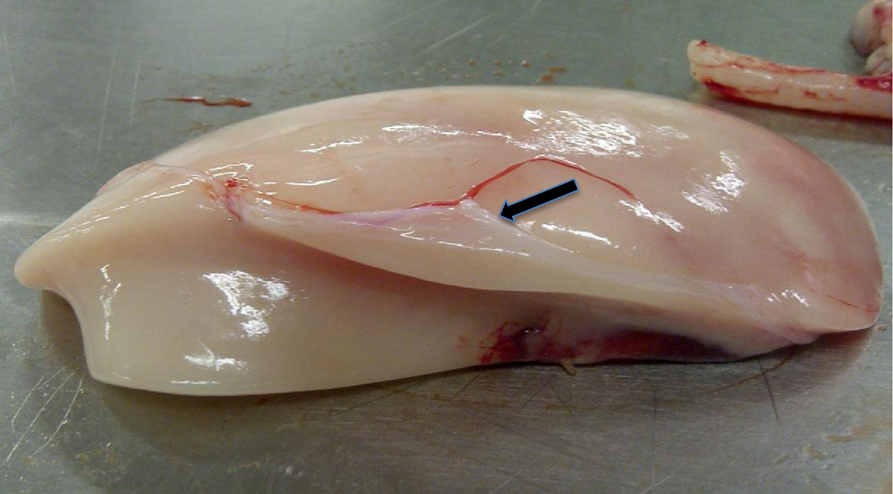
Gross structure of the abdominal fat body of the Nile crocodile. Note the white fibrous connective tissue capsule (black arrow)

**Fig. 4:**
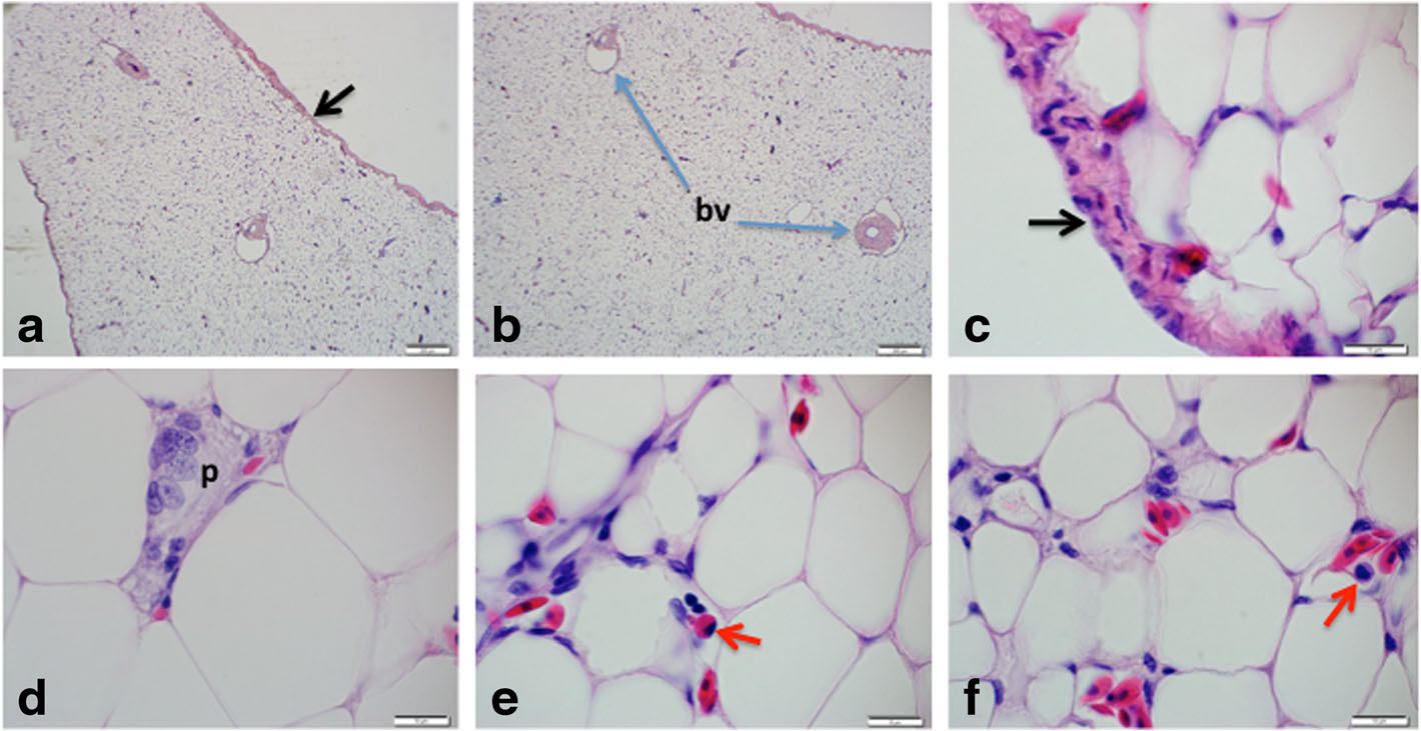
Histology (H and E) of the abdominal fat body, showing the fibrous connective tissue capsule (black arrows), blood vessels (bv) deep in the parenchyma, preadipocytes (p) and inflammatory cells, eosinophil and lymphocytes (**e**) and monocytes (**f**) (red arrows). Bars represent magnification at scales **a** & **b**, 200 μm, **c** – **f**, 10 μm

Abdominal fat body in amphibian and reptiles is a single organ that is used primarily as a fat storage organ (Fig 3). It is bounded by fibrous connective tissue capsule of collagen elastic fibres. As shown in Fig 4, the blood vessels are all located deep within the organ parenchyma with barely any close to the periphery near the capsule. The tissue is highly vascularized with several interspersed capillaries. The adipocytes are hexagonal to round in shapes with variable sizes and less compacted ECM, unlike that of the subcutaneous adipose tissue.

The ECM is large enough to accommodate blood capillaries, inflammatory cells (some of which were found wondering within the ECM, with a lot of collagen fibres and immure adipocytes (preadipocytes).

### Intramuscular adipose tissue

**Fig. 5:**
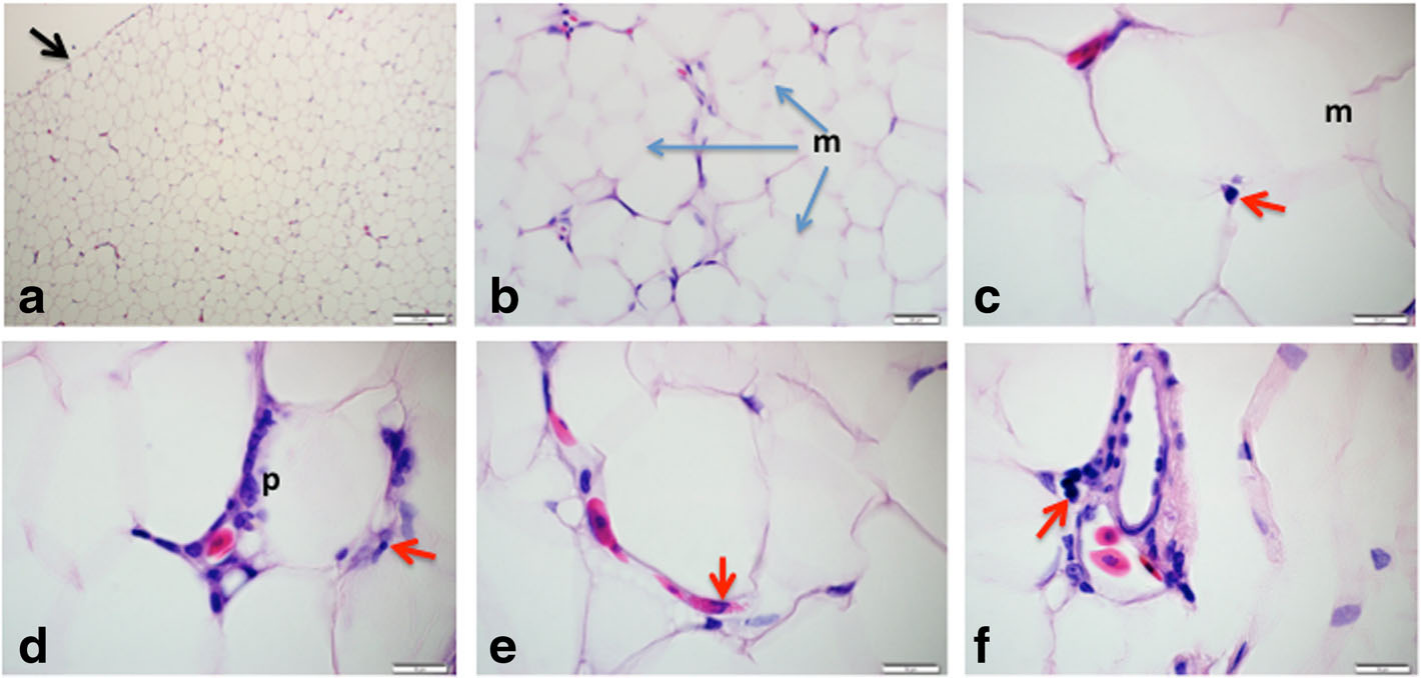
Histology of the intramuscular adipose tissue of the Nile crocodile, showing the thin fibrous connective tissue capsule – black arrow (**a**), thick collagen fibers in the ECM forming a mosaic pattern (m) in **b** and **c**. Inflammatory cells (red arrow), monocyte (**c**), small lymphocyte (**d**), eosinophil (**e**), large lymphocyte (**f**) and preadipocytes (p) are seen in the highly compressed blood vessel. Bars represent the magnifications at scales **a**, 200 μm, **b**, 20 μm, **c** – **f**, 10 μm

The external fibrous connective tissue capsule of the intramuscular adipose tissue is very thin (the thinnest of all) with sparse fibroblast (Fig. 5a). The cells (adipocytes) are tightly packed, oval to round in shape with high collagen fibre content lining the outer surface of the adipocytes in the ECM forming a mosaic pattern (Fig. 5b and c), which was not observed in other adipose tissue from other regions. Inflammatory cells are limited to the highly compressed blood capillaries (Fig. 5d and e).

### Adipose tissues in pansteatitis

**Fig. 6:**
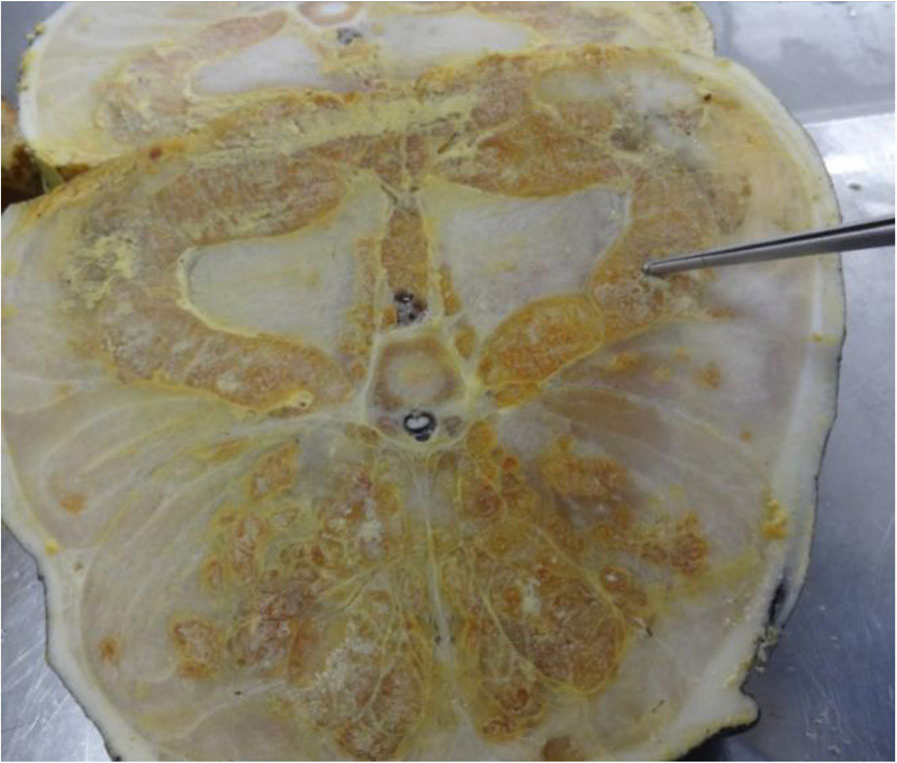
Gross presentation of pansteatitis in the intramuscular adipose tissue from the tail region of the Nile crocodile

**Fig. 7:**
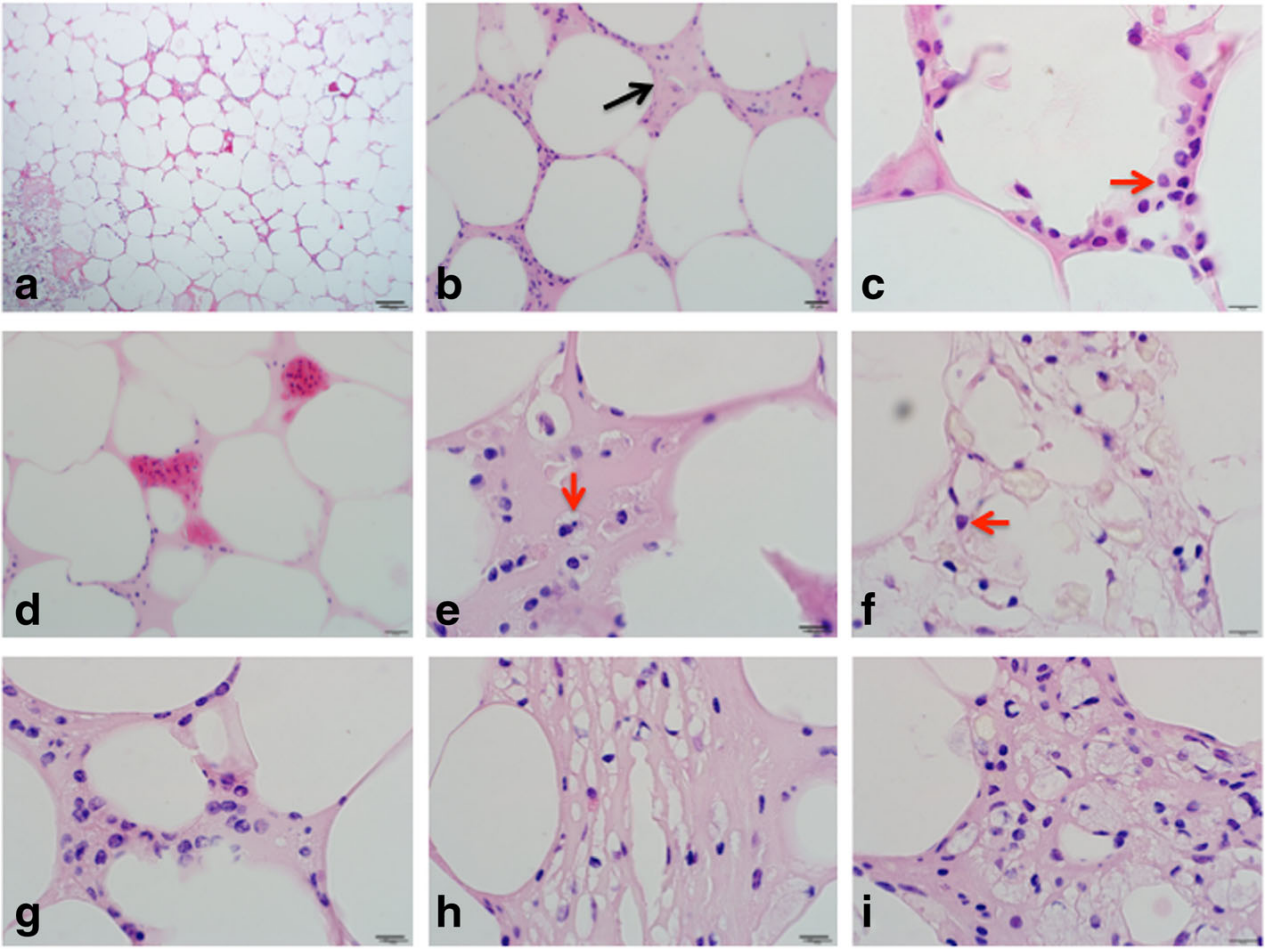
Histology of the visceral and intramuscular adipose tissue of the Nile crocodile in pansteatitis. Note the disruption of the general architecture; cellular infiltration (**a** & **b**) and expansion of the ECM with inflammatory fluid and collagen (black arrow) in slide **b** as well as congestion in the capillaries (**d**). Some plasma cells could be seen (**c**) red arrow and macrophages with foaming cytoplasm (**e**) with toxic changes in the nucleus (see the red arrow in slide **f**). Also note the presence of preadipocytes in the ECM (**g**) fibrous connective tissue lay down with fibroblast and macrophages (**h**) and more macrophages (**i**). Horizontal Bars represent magnifications at scales **a**, 100 μm, **b**, 20 μm, **c** – **i**, 10 μm

Gross presentation of pansteatitis in the intramuscular adipose tissue from the tail region of the Nile crocodile is shown in Fig. 6. Note that the skeletal muscle was not affected by the condition. Histology of the adipose tissue from the visceral and intramuscular fat from Nile crocodile that had died of pansteatitis was also examined. As shown in Fig. 7, there was a generalized disruption of the architecture and expansion of the ECM of the adipose tissue with inflammatory fluid, fibrous connective tissue and cellular infiltration of the ECM. Macrophages with foaming cytoplasm, small lymphocytes and plasma cells were the main inflammatory cells observed while fibroblast and collagen were also present in large quantities. Giant cells were also observed while macrophages with toxic changes in the nucleus could also be seen in the ECM. Some macrophages in the ECM also show severe vacuolation and accumulation of lipids.

### Transmission electron microscopy

**Fig. 8:**
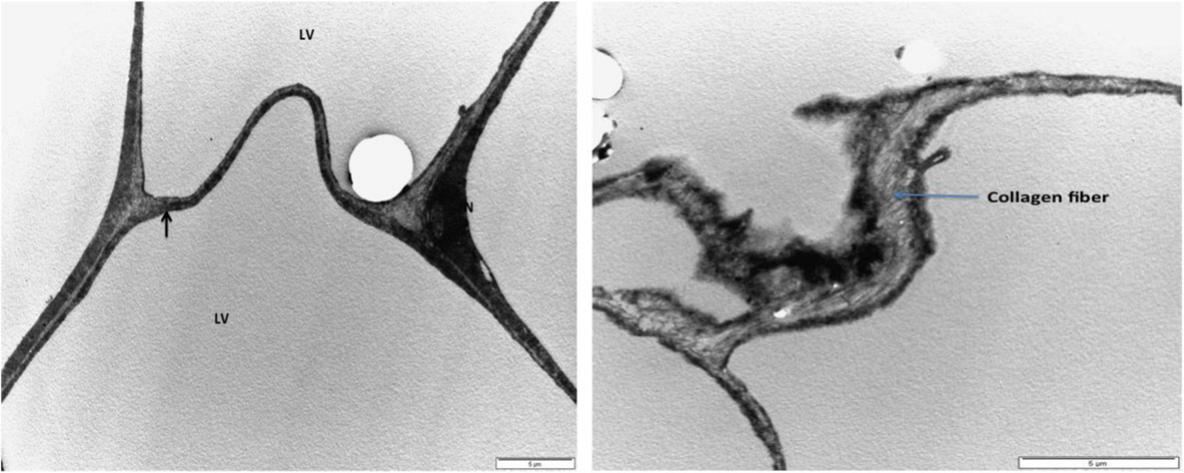
Transmission electron micrograph of the visceral adipose tissue showing the nucleus (N) and a prominent nucleolus, single lipid vacuole (LV), thin rim of cytoplasm (black arrow) and collagen fibers in the ECM. Bar represents magnification, scale 5 μm. Bar represents magnification, scale at 5 μm

**Fig. 9:**
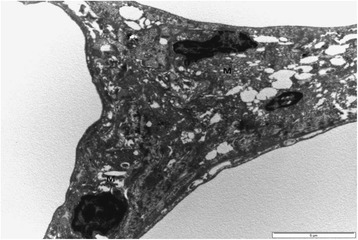
Transmission electron micrograph of the adipose tissue in pansteatitis, showing the ECM with macrophages (M) in the extracellular matrix. Bar represents magnification, scale 5 μm

Transmission electron microscope evaluation of the adipose tissue in healthy visceral, subcutaneous and intramuscular as well as pansteatitis samples was also carried out in this study. No significant regional variations were observed in the visceral, subcutaneous, abdominal fat body and intramuscular adipose tissue cells. The cells were filled with a single lipid vacuole with thin rim of cytoplasm and the nuclei displaced to the periphery (Fig. 8). The nuclei are mostly flattened with one prominent nucleolus, while the extracellular matrix shows the presence of collagen on the outer surface of the plasma membrane. Macrophages are seen in the ECM (Fig. 9).

**Fig. 10:**
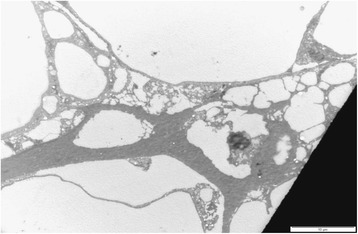
Transmission electron micrograph of the adipose tissue in pansteatitis. Note the disruption and increase in size/volume of the ECM, filled with inflammatory fluid and debris. Bar represents magnification, scale 10 μm

In a manner similar to the observation under the light microscope (H and E), the TEM of the adipose tissue in pansteatitis is devoid of regular architecture, with exaggerated ECM filled with inflammatory cell, fluid and cell debris (Fig. 10).

**Fig. 11:**
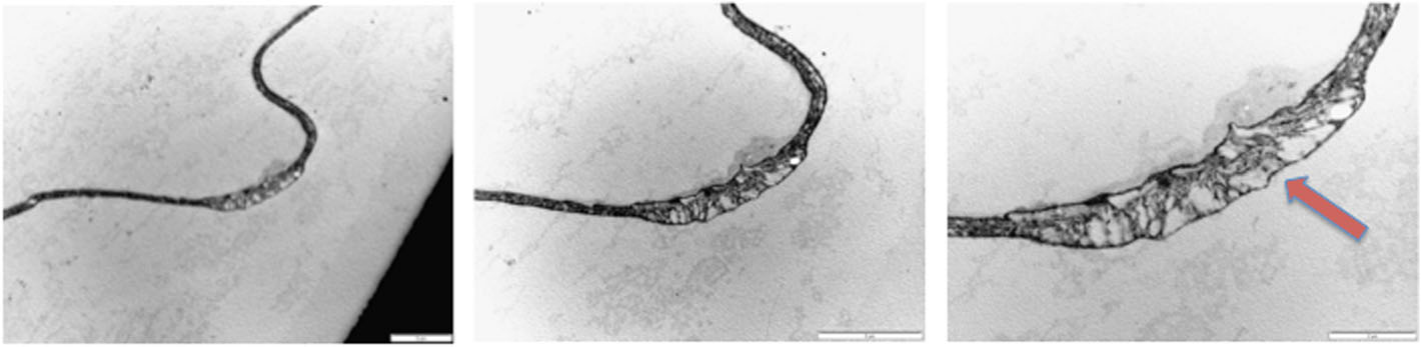
TEM of the adipose tissue showing a point of interaction between the plasma membrane of two adjacent adipocytes (red arrow). Bar represents magnification, scale 5 μm, 5 μm and 10 μm, respectively

As shown in Fig. 11, we observed a point of interaction or communication between the plasma membrane of two adjacent adipocytes that appears different from previously reported junctional complexes. It may also be an initial point of ECM expansion as inflammatory fluid begins to accumulate in the extracellular matrix.

**Fig. 12:**
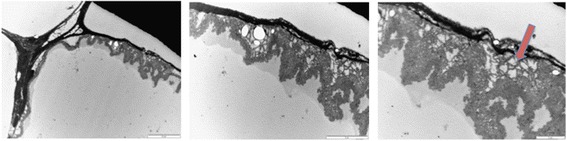
Transmission electron micrograph of the adipose tissue showing a meshwork of intracellular cytoskeleton? between the plasma membrane and the lipid vacuole (red arrow). Bar represents magnification, scale 10 μm, 5 μm and 2 μm, respectively

Similarly, we also observe the presence of a meshwork or trabeculae of intracellular myofibrils between the membrane of the lipid vacuole and the plasma membrane (Fig. 12).

### Long chain fatty acid analysis

**Fig. 13:**
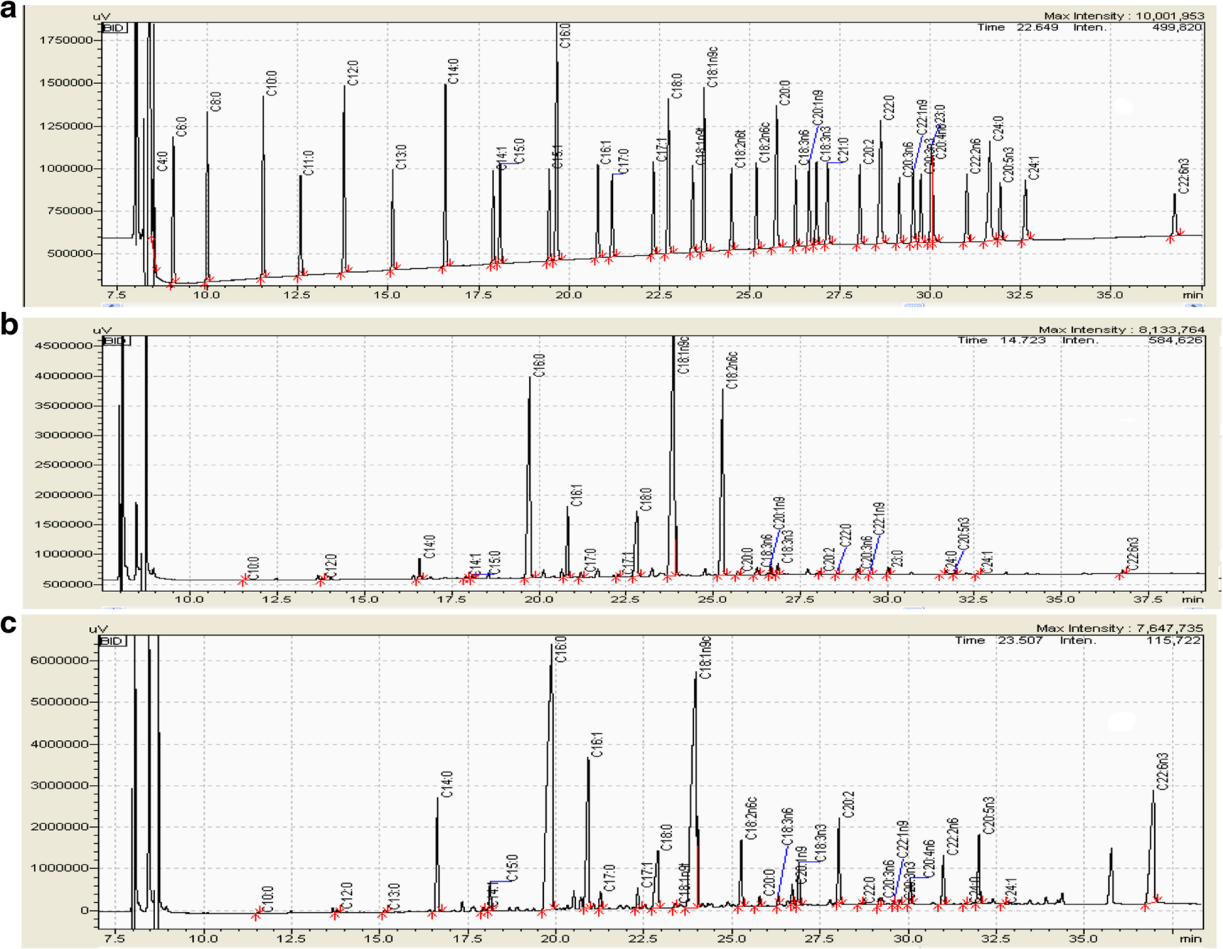
Sample of LC/MS chromatogram showing the spectrum for long chain fatty acid Composition. **a** – reference standard, **b** – healthy visceral fat and **c** – pansteatitic visceral fat

The long chain fatty acid composition of the adipose tissue was determined using LC/MS (chromatogram samples are shown in Fig 13) and analysed according to regions as well as in pansteatitis as shown in Table 1 and 2 and compared with liver samples from healthy crocodiles. Of all the 37 fatty acids in the FAME standard that was used in this study, 31 were found represented in all our samples. However, Lauric acid (C12:0), Tridecanoic acid (C13:0), cis −10- Pentadecenoic acid (C15: 1) and Linolelaidic acid (C18: 2n6t) were found in trace amounts in pansteatitis samples while Henecosanoic acid (C21:0) was found in trace amount in the liver (Table 2).

**Table 1:** Concentrations of long chain fatty acid in adipose tissue of the Nile crocodile (*Crocodylus niloticus*) with and without pansteatitis, compared with that of the liver. Values expressed as mean ± SEM in mg/dl

Fatty acids (mg/dl)		Abdominal fat body *n* = 10	Visceral fat *n* = 10	Intramuscular fat *n* = 10	Subcutaneous fat *n* = 8	Liver *n* = 10	Pansteatitis fat *n* = 10	*P* values
C13:0	Methyltridecanoate (Tridecanoic acid)	Nil	Nil	Nil	Nil	Nil	0.40 ± 0.20	
C14:0	Myristic acid (Tetradecanoic acid)	13.53 ± 1.44	14.15 ± 1.63	16.17 ± 1.63	10.96 ± 1.36	3.41 ± 1.19	126.8 ± 12.23	P < 0.0001
C14:1	Myristoleic acid (Tetradecenoic acid)	Nil	0.30 ± 0.15	1.70 ± 1.38	0.25 ± 0.16	0.62 ± 0.35	1.00 ± 0.47	ns
C15:0	Pentadecylic acid	2.80 ± 1.15	1.94 ± 0.17	2.50 ± 0.13	1.44 ± 0.17	4.10 ± 1.30	25.45 ± 2.76	P < 0.0001
C16:0	Palmitic acid (Hexadecanoic acid)	274.80 ± 20.57	301.7 ± 16.38	309.3 ± 7.82	249.2 ± 8.23	101.4 ± 11.15	741.0 ± 62.94	P < 0.0001
C16:1	Palmitoleic acid (*cis*-Δ^9^-Hexadecenoic acid)	48.47 ± 4.76	56.61 ± 3.92	55.81 ± 2.46	49.04 ± 6.99	8.56 ± 1.95	214.70 ± 27.59	P < 0.0001
C17:0	Margaric acid	2.67 ± 0.41	3.95 ± 0.35	4.03 ± 0.39	2.76 ± 0.51	1.55 ± 0.37	21.52 ± 1.82	P < 0.0001
C17:1	Heptadecenoic acid	0.03 ± 0.15	2.20 ± 0.78	0.50 ± 0.0.16	0.75 ± 0.41	Nil	18.60 ± 2.12	P < 0.0001
C18:0	Stearic acid (Octadecanoic acid)	76.86 ± 3.99	42.93 ± 14.66	96.55 ± 2.18	68.81 ± 9.94	23.34 ± 7.21	107.3 ± 10.83	P < 0.0001
C18:1n9c	Oleic acid (*cis*-Δ^9^–Octadecenoic acid)	381.3 ± 17.93	455.3 ± 30.14	471.3 ± 17.4	390.0 ± 47.11	107.4 ± 14.17	582.1 ± 66.7	P < 0.0001
C18:1n9t	Elaidic acid (*trans*-Δ^9^-Octadecenoic acid)	Nil	Nil	Nil	Nil	2.34 ± 3.49	Nil	
C18:2n6c	Linoleic acid (Methyl lineoleate)	207.3 ± 13.31	255.8 ± 14.10	249.1 ± 7.93	236.7 ± 16.85	64.88 ± 8.89	76.18 ± 11.18	P < 0.0001
C20:0	Arachidic acid (Eicosanoic acid)	0.90 ± .34	1.00 ± 0.39	1.20 ± 0.41	22.25 ± 21.42	Nil	6.47 ± 0.73	ns
C18:3n6	γ Linolenic acid	4.10 ± 1.25	4.70 ± 1.20	4.39 ± 1.15	3.34 ± 1.37	3.43 ± 1.13	6.79 ± 0.69	ns
C18:3n3	α Linolenic acid	7.87 ± 0.73	9.65 ± 0.80	10.0 ± 0.60	8.14 ± 1.07	0.90 ± 0.38	48.11 ± 8.30	P < 0.0001
C20:1n9	Eicosaenoic acid	4.32 ± 0.59	4.71 ± 0.34	4.78 ± 0.38	3.86 ± 0.64	0.50 ± 0.22	18.46 ± 3.55	P < 0.0001
C20:2	Eicosadienoic acid	1.00 ± 0.42	2.32 ± 0.59	2.03 ± 0.65	2.14 ± 0.92	0.80 ± 0.36	87.03 ± 17.78	P < 0.0001
C22.0	Behenic acid (Docosanoic acid)	0.20 ± 0.13	0.40 ± 0.22	0.50 ± 0.16	0.37 ± 0.81	1.00 ± 0.39	Nil	ns
C20:3n6	Eicosatrienoic acid	3.06 ± 0.70	4.35 ± 0.90	4.26 ± 0.93	2.94 ± 1.19	6.70 ± 0.81	8.83 ± 1.70	P < 0.01
C20:3n3	Eicosatrienoic acid	Nil	Nil	Nil	Nil	Nil	4.30 ± 0.88	
C20:4n6	Arachidonic acid	5.18 ± 0.85	6.34 ± 1.18	5.94 ± 0.97	4.72 ± 1.42	12.02 ± 2.00	18.52 ± 3.57	P < 0.0001
C24:0	Lignoceric acid (Tetracosanoic acid)	Nil	Nil	Nil	2.00 ± 1.68	Nil	47.00 ± 13.14	
C24:1	Nervonic acid (*cis*-15-tetracosenoic acid)	0.80 ± 0.20	1.00 ± 0.0	1.00 ± 0.0	0.75 ± 0.25	1.81 ± 0.24	1.20 ± 0.20	P < 0.01
C20:5n3	Eicosapentaenoic acid	5.24 ± 0.98	6.96 ± 1.59	7.88 ± 1.26	5.50 ± 1.43	2.54 ± 0.73	94.81 ± 18.64	P < 0.0001
C22:6n3	Docohexaenoic acid	8.60 ± 0.97	8.67 ± 1.55	10.03 ± 1.11	5.99 ± 1.38	3.06 ± 0.61	300.7 ± 57.57	P < 0.0001
Total Lipid		1033.0 ± 54.7	1217.0 ± 67.6	1255.0 ± 30.1	1059.0 ± 29.1	354 ± 42.3	2551 ± 289.3	P < 0.0001

**Table 2:** Long chain fatty acid composition of the adipose tissue of the Nile crocodile (*Crocodylus niloticus*) with and without pansteatitis, compared with that of the liver. Values expressed as mean ± SEM in % composition of the total lipid

Fatty acids (%)		Abdominal fat body *n* = 10	Visceral fat *n* = 10	Intramuscular fat *n* = 10	Subcutaneous fat *n* = 8	Liver *n* = 10	Pansteatitis fat *n* = 10	*P* values
C12:0	Lauric acid	Nil	Nil	Nil	Nil	Nil	0.00387 ± 0.0038	ND
C13:0	Methyltridecanoate (Tridecanoic acid)	Nil	Nil	Nil	Nil	Nil	0.00634 ± 0.0038	ND
C14:0	Myristic acid (Tetradecanoic acid)	1.30 ± 0.13^**a**^	1.16 ± 0.12^**b**^	1.22 ± 0.148^**c**^	1.00 ± 0.11^**d**^	0.77 ± 0.27^**e**^	5.10 ± 0.21^**abcde**^	*P* < 0.0001
C14:1	Myristoleic acid (Tetradecenoic acid)	0.006 ± 0.004^**a**^	Nil	0.024 ± 0.009^**b**^	0.018 ± 0.007^**c**^	Nil	0.054 ± 0.01^**abc**^	*P* < 0.0001
C15:0	Pentadecanoic acid	0.14 ± 0.02^**a**^	0.16 ± 0.01^**b**^	0.15 ± 016^**c**^	0.13 ± 0.01^**d**^	0.15 ± 0.05^**e**^	1.04 ± 0.08^**abcde**^	*P* < 0.0001
C15:1	*cis*-10-Pentadecenoic acid	Nil	Nil	Nil	Nil	Nil	0.052 ± 0.023	*P* < 0.01
C16:0	Palmitic acid (Hexadecanoic acid)	26.460 ± 0.97	24.83 ± 1.908	22.82 ± 1.91^**ab**^	23.74 ± 0.193^**c**^	29.48 ± 1.590^**a**^	30.30 ± 1.63^**bc**^	*P* < 0.0001
C16:1	Palmitoleic acid (*cis*-Δ^9^-Hexadecenoic acid)	4.67 ± 0.34^**ab**^	4.65 ± 0.15^**cd**^	4.10 ± 0.36^**ef**^	4.91 ± 0.25^**gh**^	2.14 ± 0.31^**acegi**^	8.32 ± 0.40^**bdfhi**^	*P* < 0.0001
C17:0	Margaric acid	0.27 ± 0.04^**a**^	0.33 ± 0.02^**b**^	0.31 ± 0.04^**c**^	0.25 ± 0.04^**d**^	0.37 ± 0.11^**e**^	0.84 ± 0.11^**abcde**^	*P* < 0.0001
C17:1	Heptadecenoic acid	0.022 ± 0.01^**a**^	0.0076 ± 0.026^**b**^	0.042 ± 0.014^**c**^	0.0413 ± 0.018^**d**^	Nil	0.74 ± 0.03^**abcd**^	*P* < 0.0001
C18:0	Stearic acid (Octadecanoic acid)	7.48 ± 0.21^**ab**^	7.40 ± 0.34^**cd**^	7.15 ± 0.62^**ef**^	7.22 ± 0.14^**gh**^	13.20 ± 0.97^**acegi**^	4.39 ± 0.30^**bdfhi**^	*P* < 0.0001
C18:1n9c	Oleic acid (*cis*-Δ^9^–Octadecenoic acid)	37.09 ± 0.94 ^**ab**^	37.34 ± 0.91 ^**cd**^	35.16 ± 3.14^**e**^	40.68 ± 0.98^**fg**^	30.05 ± 0.99^**aefh**^	22.92 ± 0.99^**bdegh**^	*P* < 0.0001
C18:1n9t	Elaidic acid (*trans*-Δ^9^-Octadecenoic acid)	Nil	Nil	Nil	Nil	0.67 ± 0.08^**a**^	0.02 ± 0.013^**a**^	*P* < 0.001
C18:2n6t	Linolelaidic acid	Nil	Nil	Nil	Nil	Nil	0.0026 ± 0.0026	ND
C18:2n6c	Linoleic acid (Methyl lineoleate)	20.07 ± 0.69^**a**^	21.14 ± 0.82^**b**^	18.33 ± 1.51^**c**^	19.85 ± 0.32^**d**^	18.40 ± 0.87^**e**^	2.87 ± 0.25^**abcde**^	*P* < 0.0001
C20:0	Arachidic acid (Eicosanoic acid)	0.0045 ± 0.062^**a**^	0.029 ± 0.044^**b**^	0.95 ± 0.032^**c**^	0.05 ± 0.023^**d**^	0.008 ± 0.008^**e**^	0.26 ± 0.024^**abcde**^	*P* < 0.0001
C18:3n3	α Linolenic acid	0.76 ± 0.06^**ab**^	0.79 ± 0.05^**cd**^	0.76 ± 0.08^**ef**^	0.78 ± 0.07^**gh**^	0.20 ± 0.07^**acegi**^	1.75 ± 0.21^**bdfhi**^	*P* < 0.0001
C18:3n6	γ Linolenic acid	0.39 ± 0.13^**a**^	0.38 ± 0.10^**b**^	0.34 ± 0.96^**c**^	0.37 ± 0.13^**d**^	1.50 ± 0.33^**abcde**^	0.28 ± 0.026^**e**^	*P* < 0.0001
C20:1n9	Eicosaenoic acid	0.41 ± 0.05^**ab**^	0.40 ± 0.03^**cd**^	0.35 ± 0.04^**e**^	0.32 ± 0.04^**f**^	0.12 ± 0.04^**acg**^	0.75 ± 0.13^**bdefg**^	*P* < 0.0001
C20:2	Eicosadienoic acid	0.07 ± 0.05^**a**^	0.18 ± 0.04^**b**^	0.15 ± 0.05^**c**^	0.11 ± 0.03^**d**^	0.20 ± 0.09^**e**^	3.18 ± 0.38^**abcde**^	*P* < 0.0001
C21:0	Heneicosanoic acid	Nil	Nil	Nil	Nil	0.004 ± 0.004	Nil	ND
C22.0	Behenic acid (Docosanoic acid)	0.017 ± 0.01^**a**^	0.023 ± 0.012^**b**^	0.039 ± 0.013^**c**^	0.018 ± 0.009^**d**^	0.27 ± 0.012^**abcde**^	0.002 ± 0.002^**e**^	*P* < 0.01
C20:3n6	Eicosatrienoic acid	0.30 ± 0.07	0.35 ± 0.07	0.33 ± 0.08	0.34 ± 0.11	1.89 ± 0.18^**abcde**^	0.39 ± 0.08	*P* < 0.0001
C22:1n9	Erucic acid	0.001 ± 0.001^**a**^	0	0.002 ± 0.001	0	0	0.009 ± 0.004^**a**^	*P* < 0.01
C20:3n3	Eicosatrienoic acid	Nil	Nil	Nil	Nil	Nil	0.15 ± 0.03	ND
C20:4n6	Arachidonic acid	0.51 ± 0.08^**a**^	0.52 ± 0.08^**b**^	0.46 ± 0.08^**c**^	0.53 ± 0.11^**d**^	3.59 ± 0.61^**abcde**^	0.67 ± 0.09^**e**^	*P* < 0.0001
C22:2n6	Docosadienoic acid	0	0	0.003 ± 0.003^**a**^	0.008 ± 0.008^**ab**^	0	1.90 ± 0.17	*P* < 0.0001
C24:0	Lignoceric acid (Tetracosanoic acid)	0.004 ± 0.003^**a**^	0.002 ± 0.001^**b**^	0.004 ± 0.002^**c**^	0	0.10 ± 0.05^**abcd**^	0.0003 ± 0.0003^**d**^	*P* < 0.01
C24:1	Nervonic acid (*cis*-15-tetracosenoic acid)	0.03 ± 0.01^**a**^	0.03 ± 0.01^**b**^	0.04 ± 0.01^**c**^	0.03 ± 0.10^**d**^	0.60 ± 0.11^**abcde**^	0.02 ± 0.008^**e**^	*P* < 0.0001
C20:5n3	Eicosapentaenoic acid	0.50 ± 0.09^**a**^	0.57 ± 0.13^**b**^	0.62 ± 0.12^**c**^	0.54 ± 0.11^**d**^	0.63 ± 0.18^**e**^	3.46 ± 0.42^**abcde**^	*P* < 0.0001
C22:6n3	Docohexaenoic acid	0.83 ± 0.09^**a**^	0.71 ± 0.12^**b**^	0.76 ± 0.12^**c**^	0.60 ± 0.11^**d**^	0.87 ± 0.16^**e**^	10.98 ± 1.28^**abcde**^	*P* < 0.0001
Total Lipid		20.13 ± 1.01^**abc**^	22.02 ± 0.91^**de**^	23.25 ± 0.54^**afgh**^	18.96 ± 0.46^**fij**^	1.24 ± 0.17^**bdgik**^	10.80 ± 0.82^**cehjk**^	*P* < 0.0001
Total Saturated Lipids		4.77 ± 1.04	4.53 ± 0.97	3.97 ± 0.88	4.32 ± 1.01	4.94 ± 1.03	4.66 ± 1.00	ns
Total Monounsaturated Lipids		6.03 ± 1.55	6.07 ± 1.55	5.67 ± 1.52	7.70 ± 2.17^**a**^	5.60 ± 1.44	3.65 ± 0.78^**a**^	ns
Total Polyunsaturated Lipids		2.93 ± 0.73	3.08 ± 0.78	2.97 ± 0.78	2.57 ± 0.73	3.51 ± 0.70	2.56 ± 0.34	ns
Total Omega 3		0.70 ± 0.05^**a**^	0.69 ± 0.06^**b**^	0.69 ± 0.08^**c**^	0.63 ± 0.06^**d**^	0.57 ± 0.10^**e**^	4.08 ± 0.74^**abcde**^	*P* < 0.0001
Total Omega 6		5.32 ± 1.38	5.60 ± 1.45	3.89 ± 1.07	4.09 ± 1.31	6.88 ± 1.25^**a**^	1.22 ± 0.16^**a**^	*P* < 0.01
Total Omega 9		12.50 ± 3.24	12.58 ± 3.27	11.84 ± 3.22	20.50 ± 5.23^**ab**^	10.28 ± 2.62^**a**^	5.93 ± 1.59^**b**^	*P* < 0.01

Values with the same superscripted alphabets are significantly different *P* < 0.001

ND means not determined

ns means not significant

### Regional variation in the fatty acid composition of adipose tissue

As shown in Table 2, Myristoleic acid (C14: 1) was found only in the abdominal fat body, intramuscular and subcutaneous fat as well as in pansteatitis samples. It was not detected in the visceral fat and the liver. Palmitic acid (C16:0) was found to be significantly higher (P < 0.001) in the liver than the composition in the intramuscular fat, while Elaidic acid (C18: 1n9t) was only found in the liver and pansteatitis, with the composition being significantly higher (P < 0.001) in the liver than that of the pansteatitis fat. In a similar manner, Erucic acid (C22: 1n9) was found only in the abdominal fat body, intramuscular fat and in pansteatitis samples - which was significantly higher than the others (P < 0.01), none was found in the visceral fat, subcutaneous fat and in the liver. Docosadienoic acid (C22: 2n6) was also absent in the abdominal fat body, visceral fat and the liver while trace amount was found in the intramuscular and subcutaneous fats. It was however found in significant amount in the pansteatitis fat and the composition was higher (P < 0.01) than those of the intramuscular and subcutaneous fats. Finally, significant amount of Lignoceric acid (C24:0) was found in the liver with its percentage composition in the abdominal fat body, visceral fat, intramuscular and pansteatitis fat being significantly lower (P < 0.01) than that of the liver, while none was found in subcutaneous fat.

**Fig. 14:**
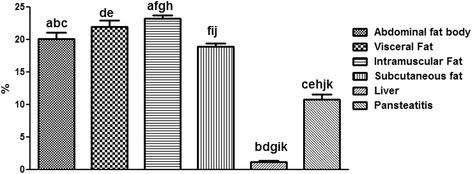
Percentage total lipid composition in healthy liver and the adipose tissue of the Nile crocodile with and without pansteatitis. Values presented as mean ± sem. Values with the same superscript alphabets are significantly different, *P* < 0.0001

**Fig. 15:**
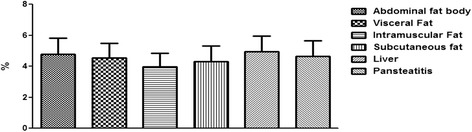
Percentage total saturated fatty acid in healthy liver and the adipose tissue of the Nile crocodile with and without pansteatitis. Values presented as mean ± sem. Values with the same superscript alphabets are significantly different, *P* < 0.0001

As shown in Fig. 14, the intramuscular fat has the highest content of fatty acid; it was significantly higher (P < 0.001) than the total fatty acid in the abdominal fat body, liver and pansteatitis samples. While the liver has the lowest long change fatty acid per unit mass and in percentages. Surprisingly however, the total percentage fatty acid composition in pansteatitis was lower than that of the healthy adipose tissue storage in the crocodile i.e., the abdominal fat body, visceral, intramuscular and subcutaneous adipose tissue storage. It was however higher (P < 0.0001) than that of the liver composition. The total saturated fatty acid, however, did not show any significant variation across the adipose tissue types and even in the liver (Fig 15).

### Long chain fatty acid in pansteatitis

As can be observed in Table 2, with the exception of Heneicosanoic acid, (C21:0), all the long chain fatty acid that were found in adipose tissue in this study are represented in the pansteatitis samples and the percentage composition of each being several folds higher than those of the healthy adipose tissues and liver, except in certain instances where the composition in pansteatitis was considerably lower than those of the healthy tissues. These include stearic acid (C18:0), Oleic acid (C18:1n9c), Linoleic acid (C18:2n6c), γ-Linoleic acid (C18:3n6) that were observed to be significantly lower (*P* < 0.001) in pansteatitis than in the other adipose tissues and the liver; while Elaidic acid (C18: 1n9t), Behenic acid (C22:0), Eicosatrienoic acid (C20:3n6), Arachidonic acid (C20:4n6), Lignoceric acid (C24:0) and Nervonic acid (C24:1) composition of the pansteatitis samples were lower than those in the liver. On the contrary, therefore, the levels/composition of Myristic acid (C14:0), Pentadecanoic acid (C15:0), Palmitic acid (C16:0), Palmitoleic acid (C16:1), Heptadecanoic acid (C17:0), Linolenic acid (C18;3n3), Arachidic acid (C20:0), Eicosaenoic acid (C20:1n9), Eicosadienoic acid (C20:2n6) as well as Erucic acid (C22:1n9), Docosadienoic acid (C22:2n6), Eicosapentaenoic acid (C20:5n3) and Docosahexaenoic acid (C22:6n3) in the pansteatitic fat were found to be significantly higher than those of the healthy adipose tissue from the abdominal fat body, visceral, intramuscular and subcutaneous fats (Table 1 and 2).

**Fig. 16:**
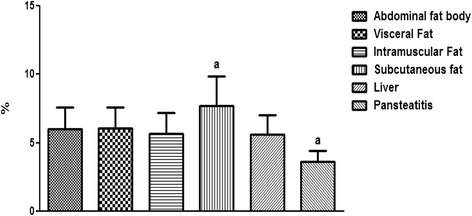
Percentage total monounsaturated fatty acid in healthy liver and the adipose tissue of the Nile crocodile with and without pansteatitis. Values presented as mean ± sem. Values with the same superscript alphabets are significantly different, *P* < 0.0001

**Fig. 17:**
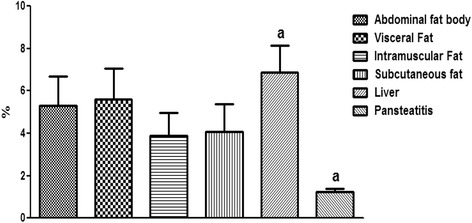
Percentage total polyunsaturated fatty acid in healthy liver and the adipose tissue of the Nile crocodile with and without pansteatitis. Values presented as mean ± sem. Values with the same superscript alphabets are significantly different, *P* < 0.0001

**Fig. 18:**
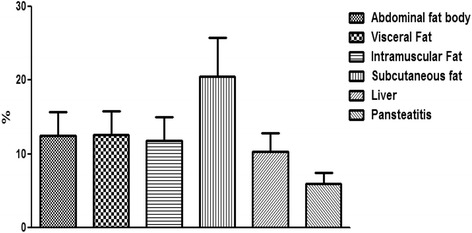
Percentage total omega 3 fatty acid in healthy liver and the adipose tissue of the Nile crocodile with and without pansteatitis. Values presented as mean ± sem. Values with the same superscript alphabets are significantly different, P < 0.0001

**Fig. 19:**
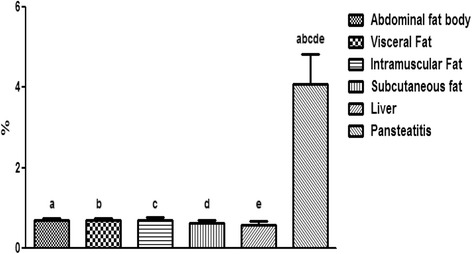
Percentage total omega 6 fatty acid in healthy liver and the adipose tissue of the Nile crocodile with and without pansteatitis. Values presented as mean ± sem. Values with the same superscript alphabets are significantly different, *P* < 0.0001

**Fig. 20:**
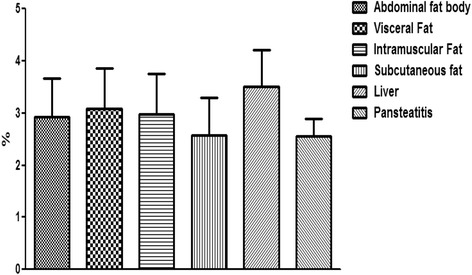
Percentage total omega 9 fatty acid in healthy liver and the adipose tissue of the Nile crocodile with and without pansteatitis. Values presented as mean ± sem. Values with the same superscript alphabets are significantly different, *P* < 0.0001

Furthermore and contrary to our expectation, the percentage total lipid in the pansteatitis samples was found to be 10.80 ± 0.83%, which was significantly lower (P < 0.0001) than the total percentage lipid of 20.13 ± 1.01%, 22.02 ± 0.91%, 23.25 ± 0.54% and 18.96 ± 0.46% in the abdominal fat body, visceral, intramuscular and subcutaneous fats respectively (Fig. 14). The lowest fatty acid composition percentage per tissue mass was however found in the liver at 1.24 ± 0.17%, which was significantly lower than any of the adipose tissue samples mentioned earlier. Although, total saturated fatty acid were similar in all samples (Fig. 15), the total monounsaturated (Fig. 16), total omega 6 and 9 fatty acids (Figs. 17 and 18) were lower in pansteatitis while total omega 3 fatty acid was higher significantly in the pansteatitis sample than those of the other adipose tissue and the liver (Fig. 19). The total polyunsaturated fatty acid was also lower in the pansteatitis samples than in the other samples evaluated, although non-significantly (Fig. 20).

## Discussion

### Histomorphology and ultrastructure

Comparative evaluation of the adipose tissues in the Nile crocodile showed considerable regional variation in the histological structure. Visceral adipose tissue was found to be lobated with the fibrous connective tissue capsule extending in various degrees into the parenchyma, but without total division into lobules, whereas the subcutaneous adipose tissue was observed to be lobular or lobulated. The abdominal fat body did not show any penetration of the connective tissue capsule into the parenchyma, thus forming a single non-divisible lipid storage organ irrespective of the size. The intramuscular adipose tissue is characterized by the enormous accumulation of collagen fibre in the extracellular matrix in a mosaic like appearance that is found only in this tissue. This is a type 3 or fibrous adipose tissue as enumerated by Sbarbati et al. [30], they are reportedly found in areas that are more subject to mechanic stress, hence a more developed periadipocyte collagens basket. Based on this collagen composition, visceral adipose as well as the abdominal fat body can be grouped as type 2 or stromal white adipose tissue because of the less collagen than that of the intramuscular adipose tissue, while the subcutaneous adipose tissue with the least amount of collagen could be described as storage or type 1 adipose. Although, this division may be influenced by the age of the subject, as age affects lay down of collagen [30], it inadvertently affects the development of the adipose tissue involved as it influences the differentiation of the preadipocytes, proliferation of adipocytes and subsequent accumulation of lipids through the influence of membrane anchored metalloproteinase (MT1-MMP) in close association with the collagen [8]. These authors were able to demonstrate that differentiation and maturation of adipocytes requires an MT1-MMP-mediated proteolysis that modulates pericellular collagen rigidity especially Type I collagen in the control of adipogenesis.

It has also been reported that MT1-MMP suppressed expression of many of the adipogenic transcription factors including PPARγ (peroxisome proliferator activated receptor γ), SREBP-1 (sterol regulatory element binding protein-1), C/EBP (CCAAT/enhancer binder protein family), leptin and UCP1 in the mice [8]. Thus, the presence of collagen in the ECM of the adipose tissues, apart from providing support are also involved in signalling and modulation of adipogenesis and maturation as well as excessive lipid accumulation and subliminal inflammation associated within it [20]. In another twist to the roles of collagen in adipogenesis and adipocytes metabolism, Khan et al., [20] though reported the presence of collagen I and IV in various adipose tissues in mice, maintained that collagen IV is predominant and is responsible for the adipogenesis and excessive accumulation of lipids in adipocytes thereby reducing obesity in healthy mice or mitigating the metabolic effects of obesity and inflammation in ob/ob knockout mice. Identification and probable characterization of the various collagen types in crocodile however, to directly establish their roles in adipogenesis and metabolism is a subject of future studies in this animal species.

In pansteatitis however, we observed a considerable lay down of fibrous connective tissue (fibrosis), lymphocytic and macrophage infiltration a hallmark of chronic granulomatous inflammation. Increased collagen/fibrosis and subsequent development of chronic inflammation in the adipose tissue associated with increased fat diet has been well documented in literature, especially in humans and other model animals [14, 32]. This has been attributed to several mechanisms, ranging from hypoxia induced HIF1α (hypoxia-inducible factor 1α) as it facilitates the disruption of collagen crosslinking, stabilization and subsequent development of inflammation and fibrosis in adipose tissue [11, 32] and other tissue types in the body [31]; up regulation of inflammatory adipokines and cytokines and activation of the macrophages in the ECM [13, 25]. The distribution of collagen in the ECM might thus contribute to the degree of inflammation in pansteatitis, because the degree and severity of pansteatitis appeared to be more in adipose tissue with high collagen content in healthy subject since abdominal fat body was observed to show little or no signs of inflammation in the affected crocodiles [24].

The distribution and density of blood vessels in the adipose tissue in the crocodile also showed some degrees of variations in the healthy adipose tissue, which could be used to differentiate adipose tissue from different regions. For example, the most vascularized adipose tissue of the Nile crocodile as observed in this study is the abdominal fat body with several large vessels interspersed deeply in the parenchyma and more capillaries in the ECM while the visceral adipose tissue has the least blood vessel density within the parenchyma with most of the major vessels located close to the capsule. This might be an important factor as adipose tissue lay down increases either with increase nutrition only or in combination with increase in age as the rate of angiogenesis and tissue perfusion might not be able to catch up with the demand by the adipose tissue. Thus resulting in hypoxia and up regulation of inflammatory genes including IL-6, macrophage inflammation factor 1 (MIF1) through HIF1α induction [32].

Sudden exposure of the crocodiles to fish and overfeeding as a result of fish die offs in and along the Olifants River gorge, Kwazulu Natal Province where the condition was identified [2] might have led to adipose tissue hypertrophy, serving as a trigger for chronic inflammation in the affected animals. This has been observed in acute overfeeding with high caloric energy balance resulting in oxidative stress in the adipose tissue [4]. More chronic over nutrition however is more favourable to inflammation and fibrosis in adipose tissue as a result of persistent endoplasmic reticulum (ER) stress and increased macrophage chemoattractant protein 1 (MCP-1), a proinflammatory cytokine that has been isolated in adipose tissue. It is found in association with hypertrophic adipocytes and subsequent macrophage infiltration, activation and production of inflammatory cytokines just after 3 weeks of high fat diet in mice [23].

Apart from the various predisposing factors such as reduced tissue perfusion especially, deep within the parenchyma, presence of mononuclear cells and collagen in the ECM, we also observed the presence of toxic changes within the nuclei of some of the mononuclear phagocytic cells in pansteatitis samples (Fig. 5f). Toxic nuclei as the name implies are changes observable in blood cells exposed to chemical contaminants resulting in clumping of chromatins in the nucleus. This has been used in the diagnosis of poisoning. The presence of these toxic changes may be an indicator that the animals might have been exposed to toxic chemicals or compounds in the environment, more so that the presence of heavy metal in AMD water and POPs (persistent organophosphates) contamination has been reported in Olifants River and their presence correlated with the development of pansteatitis in the Tilapia [27]. Acid mine drainage water and POPs have also been reported to persist in the environment especially in algae and phytoplankton in the area [26].

### Lipids analysis

Fatty acid methyl ester (FAME) analysis of the long chain fatty acid of the adipose tissue in healthy adipose tissue of the Nile crocodile also showed some degree of regional variation in the lipid composition, though more considerably differences were observed when compared with lipids in the liver and pansteatitis samples. For example, Myristoleic acid (C14:1) was absent in the visceral fat and in the liver, Erucic acid (C22:1n9) was absent in visceral and subcutaneous fats and in the liver while Oleic acid (C18:1n9c) was found only in the liver and pansteatitis fat with the percentage composition of oleic acid in the liver being significantly higher than that of pansteatitis. Cis pentadecenoic acid (C15:1), Linolelaidic acid, Eicosatrienoic acid (C20:3n3) were only found in pansteatitis while Heneicosanoic acid (C21:0) was found only in the liver.

Myristoleic acid (C14:1), or 9-tetradecenoic acid, is an omega-5 fatty acid with 14 carbon atoms and a double bond. It is synthesized from myristic acid (C14:0) by the enzyme delta-9 desaturase. It is found in extract of the sea cucumber, (Cucumaria frondosa) with potent anticancer activity and lipoxygenase inhibitory activity [9]. It was also reported as the cytotoxic component of the extract of Serenoa repens responsible for apoptosis and necrosis in LNCaP cells. It has thus been developed as a tool in the treatment of prostate cancer [18]. Myristoleic acid and its derivative cetyl myristoleate, have also been patented in relieving the pain of rheumatoid arthritis and osteoarthritis [15, 22]. This compound being a cytotoxic compound that is capable of inducing apoptosis and necrosis, begs the question: could the presence of myristoleic acid in healthy intramuscular and subcutaneous fat as well as abdominal fat body be a risk factor in tissue damage in pansteatitis as the composition increases in pansteatitis? It is also not known whether it is being extracted in crocodile fat for the purpose of anticancer and antiarthritis therapy.

The use of crocodile oil though, has been documented in traditional healing in various ethnic groups in Africa, including South Africa, Madagascar and even China [5]. Antimicrobial effects and anti-inflammatory effects of crocodile oil has also been reported [5], but there is paucity of information in literature on the use of crocodile fats. Blood extracts of Crocodylus siamensis has also been reported to show considerable anti-inflammatory activities [21]. These authors observed anti-inflammatory effect of crocodile blood extracts by examining their inhibitory effects on pro-inflammatory mediators in lipopolysaccharide (LPS)-stimulated murine macrophage RAW 264.7 cells. They further reported that both plasma and crude leukocyte extract significantly inhibited the anti-inflammatory Nitrogen oxide (NO) production and exhibited cytotoxicity in all the tested concentrations.

We observed that although, the percentage composition of many saturated and monounsaturated fatty acid as well as some polyunsaturated fatty acid was higher in pansteatitis than in healthy adipose tissues, the percentage composition of all the fatty acid with C18 backbone (from stearic acid (C18:0), Oleic acid (C18:1n9c), Elaidic acid (C18:1n9t), Linoleic acid (C18:2n6c), Linolelaidic acid (C18:2n6t), to γ Linolenic acid (C18:3n6) except Linolenic acid (C18:3n3)) were lower in the pansteatitis samples than in the liver and all the healthy adipose tissue samples, irrespective of the region. Arachidonic acid C20:4n6) was also lower. This shows that the crocodiles with pansteatitis, though has large chunk of adipose tissue lack the major essential fatty acids, and they are instead laden with EPA and DHA, (Eicosapentenoic acid and Docosahexaenoic acid) found almost exclusively in high fat fish and marine animals, which are precursors for the synthesis of lipid derived modulators of cell signaling and inflammatory cytokines. They are also the main substrate for cyclooxygenases, lipoxygenases, Cytochrome P450 mono-oxygenases that give rise to the production of signalling molecules xincluding leukotrienes, prostanoids, thromboxanes that are implicated in various biological processes such as inflammation to PO regarding the new manuscript provided in CORRECTION.zip on how to proceed.error parsing due to changing in author group and chemotaxis [1]. This, coupled with high concentration of C18 desaturated lipids such as Stearic and Oleic acid (Table 1), which are part of the hallmark of obesity and metabolic syndrome in higher animals including humans and strong inducer of inflammation in the adipose tissue of obese animals [34] appears to be predisposing factors to pansteatitis in the crocodile.

Although some authors have hypothesized some degree of anti-inflammatory roles to EPA and DHA, especially against lipopolysaccharide (LPS) induced activation of Toll-like receptor 4 (TLR4) in the downstream activation and pro-inflammatory roles of TNFα and IL-6, [16] the overwhelming effects of other saturated fatty acid and Myristoleic acid to induce persistent inflammation in pansteatitis may have subsisted because of their high concentration per unit tissue (Table 1).

Palmitic and palmitoleic acids were also significantly higher in pansteatitis than in healthy adipose tissue. Palmitic acid, palmitate or Hexadecanoic acid (C16:0) has been reported to induce endoplasmic reticulum (ER) stress and subsequently autophagy and apoptosis in 3 T3-L1 adipocyte cell lines by increasing the levels of immunoglobulin binding protein – BiP (an ER stress marker), activation transcription factor 4 (ATF4) and C/EBP homologous protein (CHOP), c-Jun N-terminal kinase (JNK) and other promoters of apoptosis and autophagy [35]. This indicates that an exceptional increase in the levels of palmitate as observed in the present study in pansteatitis may not only be a predisposing factor for inflammation but also for apoptosis and autophagy in the affected adipose tissue.

On a final note, percentage of total lipid composition in the pansteatitis samples was lower than that of the healthy adipose tissue in the crocodile, the major parts being taken over by fibrous connective tissue in the chronic inflammation, this may also make the fatty acid unavailable for mobilization for oxidation as an energy source contributing to stiffness and rigidity of the adipose tissues including intramuscular adipose tissue of the tail.

## Conclusion

Adipose tissue, due to its sensitivity to lipid peroxidation by oxidative stress, low-grade/subliminal inflammation, which can affect the whole system resulting in metabolic syndrome, and other complications, persistent mononuclear infiltration and fibrosis is a good candidate in waiting for potential trigger that would tilt the balance of the health status, not only in lower vertebrates, but also in mammals and humans. And this trigger could be a simple change in the energy balance, changes in environment or exposure to environmental contaminants.

Molecular characterization of the adipose tissue in the Nile crocodile and differential expression of inflammatory and apoptosis genes in healthy and pansteatitis adipose tissue are currently being investigated in our laboratory. This will serve as a platform for further studies especially in controlled environment that may be needed to study each of the potential predisposing factors as contributors to the pathophysiology of pansteatitis that have been identified in our present study.
